# Integrated entropy-based approach for analyzing exons and introns in DNA sequences

**DOI:** 10.1186/s12859-019-2772-y

**Published:** 2019-06-10

**Authors:** Junyi Li, Li Zhang, Huinian Li, Yuan Ping, Qingzhe Xu, Rongjie Wang, Renjie Tan, Zhen Wang, Bo Liu, Yadong Wang

**Affiliations:** 1 School of Computer Science and Technology, Harbin Institute of Technology (Shenzhen), Shenzhen, Guangdong, 518055 China; 2 School of Computer Science and Technology, Harbin Institute of Technology, Harbin, Heilongjiang, 150001 China; 3 CAS Key Laboratory of Computational Biology, CAS-MPG Partner Institute for Computational Biology, Shanghai Institute of Nutrition and Health, Shanghai Institutes for Biological Sciences, University of Chinese Academy of Sciences, Chinese Academy of Sciences, Shanghai, 200031 China

**Keywords:** Information entropy, Generalized topological entropy, DNA sequences, Exon and intron prediction, Genomic signal processing

## Abstract

**Background:**

Numerous essential algorithms and methods, including entropy-based quantitative methods, have been developed to analyze complex DNA sequences since the last decade. Exons and introns are the most notable components of DNA and their identification and prediction are always the focus of state-of-the-art research.

**Results:**

In this study, we designed an integrated entropy-based analysis approach, which involves modified topological entropy calculation, genomic signal processing (GSP) method and singular value decomposition (SVD), to investigate exons and introns in DNA sequences. We optimized and implemented the topological entropy and the generalized topological entropy to calculate the complexity of DNA sequences, highlighting the characteristics of repetition sequences. By comparing digitalizing entropy values of exons and introns, we observed that they are significantly different. After we converted DNA data to numerical topological entropy value, we applied SVD method to effectively investigate exon and intron regions on a single gene sequence. Additionally, several genes across five species are used for exon predictions.

**Conclusions:**

Our approach not only helps to explore the complexity of DNA sequence and its functional elements, but also provides an entropy-based GSP method to analyze exon and intron regions. Our work is feasible across different species and extendable to analyze other components in both coding and noncoding region of DNA sequences.

**Electronic supplementary material:**

The online version of this article (10.1186/s12859-019-2772-y) contains supplementary material, which is available to authorized users.

## Background

Research on Deoxyribonucleic acid (DNA) is a key content and important foundation in biological and life science studies [1, 2]. Functional DNA elements such as genes and noncoding elements are composed of four nucleotides: adenine (A), cytosine (C), guanine (G) and thymine (T). Their functions are basically decided by order of nucleotides. Essential part of genomic sequence analysis is to identify functional elements and their positions in DNA sequence [3–8], which is a basis for further research on target genes and plays a vital role in species evolution studies.

Information theory is a science which studies the measurement, transmission, exchange and storage of information. Genetics information is supposed to follow the general law of information storage and communication. Therefore, information theory [9, 10] method is a feasible way to analyze genetic information [6, 11–13]. As a measure of information complexity, information entropy was first proposed by Shannon in 1948 [10]. It is reasonable to analyze the genome sequence based on information entropy methods. For example, there are different conserved and correlated loci on the DNA sequence and their randomness leads to various information entropy values. Based on the theory of information entropy, people can quantitatively describe the complexity of given sequences and categorize these sequences according to their complexity.

In past decade, entropy-based quantitative analysis methods facilitated calculating sequence complexity and analyzing underlying connections between genetic elements. For instance, the Shannon metric entropy is used to calculate the genomic DNA sequence of different organisms [14] and the Renyi entropy is mainly applied to evaluate randomness of the DNA sequence [15]. The diffusion entropy can be used to analyze the complexity of promoter region of the human genome [5]. Topological entropy [16, 17] and generalized topological entropy [17] are able to analyze the finite length DNA sequence [10, 13, 18, 19]. These genomic sequence analysis methods based on information entropy have obtained a series of research results. Recently, genetic information is generated exponentially with the development of next-generation technology, which puts forward higher requirements for entropy-based quantitative methods.

Genomic signal processing (GSP), which is based on digital signal processing (DSP), has been widely applied in DNA sequence studies in recent years [20–28]. In general, four nucleotides T, C, G and A are converted to corresponding numerical values and the whole sequence is presented by numerical sequence. Then the numerical sequences are analyzed by the algorithms in accordance with various purposes. Different GSP methods have their own rules of conversion and algorithms. And they are used in genetic sequence comparison [26, 27, 29], sequence alignment [30] and gene prediction [22, 31–33]. Specifically, discrete Fourier transform [34], short-time Fourier transform [22] and singular value decomposition (SVD) [31–33] have been effectively used to predict exon locations.

In this study, we designed an integrated entropy-based analysis approach, which involves modified topological entropy calculation, genomic signal processing (GSP) method and singular value decomposition (SVD) [32, 33], to investigate exons and introns in DNA sequences. We optimized and implemented the topological entropy and the generalized topological entropy to calculate the complexity of DNA sequences, highlighting the characteristics of repetition sequences. We compared the difference between digitalizing values of exons and introns, and found that a significance level of difference between them is improved with our optimized entropy calculation. After we converted DNA sequences to numerical topological entropy values, we applied SVD method to effectively investigate exon and intron regions on single gene sequences from five species. Our integrative analysis approach is also extendable to study other elements in both coding and noncoding region of DNA sequences.

## Methods

### Data sets

Data sets of exon, intron and promoter for each chromosome on human genome were used in this study (Table 1). We downloaded human genome data hg38 from UCSC (http://genome.ucsc.edu/index.html) [35] and acquired sequence information of genomic elements by Galaxy (https://usegalaxy.org/) [36]. We have filtered out sequences which have length less than 200 base pairs (bp) to lower significant noise.

**Table 1:** Mean entropy value and number (in parentheses) of exons, introns and promoters on each chromosome in human genome

	Entropy (number) of exon	Entropy (number) of promoter	Entropy (number) of intron
chr1	0.9653 (18043)	0.9643 (13010)	0.9689 (42806)
chr2	0.9677 (13911)	0.9619 (9180)	0.9687 (39446)
chr3	0.9651 (11456)	0.9648 (7992)	0.9707 (32834)
chr4	0.9656 (7087)	0.9622 (5016)	0.9697 (20301)
chr5	0.9668 (8036)	0.9621 (5834)	0.9707 (22176)
chr6	0.9653 (17918)	0.9636 (12728)	0.9687 (31005)
chr7	0.9652 (8159)	0.9631 (6140)	0.9678 (22410)
chr8	0.9646 (7170)	0.9640 (5280)	0.9682 (22011)
chr9	0.9642 (7084)	0.9637 (5230)	0.9681 (19486)
chr10	0.9677 (8529)	0.9636 (6342)	0.9690 (24883)
chr11	0.9651 (10006)	0.9640 (7504)	0.9690 (23462)
chr12	0.9660 (9533)	0.9633 (6886)	0.9695 (25561)
chr13	0.9651 (3589)	0.9638 (2684)	0.9699 (10396)
chr14	0.9666 (5948)	0.9632 (4244)	0.9691 (14017)
chr15	0.9644 (6857)	0.9643 (4706)	0.9691 (18634)
chr16	0.9636 (7303)	0.9647 (5300)	0.9691 (16214)
chr17	0.9642 (10218)	0.9649 (7118)	0.9687 (2295)
chr18	0.9656 (3041)	0.9646 (2202)	0.9697 (9062)
chr19	0.9689 (15539)	0.9647 (8016)	0.9705 (25077)
chr20	0.9634 (4724)	0.9646 (3594)	0.9702 (10692)
chr21	0.9639 (2326)	0.9612 (1732)	0.9692 (7033)
chr22	0.9626 (3985)	0.9621 (2866)	0.9675 (9221)
chrX	0.9665 (6836)	0.9615 (5392)	0.9685 (14929)
chrY	0.9647 (1121)	0.9588 (1872)	0.9671 (3320)

We used a sequence ranging from 3500–10500 bp from gene AJ229040 to predict exons and introns. The sequence was downloaded from NCBI [37]. Six exon locations are marked as 3770 – 3826 bp, 4584 – 4601 bp, 4671 – 4730 bp, 4999 – 5277 bp, 5730 – 5823 bp and 6719 – 6898 bp. We also used other 15 genes from five species (human, dog, zebrafish, C. elegans and fruitfly) for exon predictions. The sequences were downloaded from Ensembl [38]. The names of these genes are listed in Additional file 1: Table S3.

### Modified topological entropy and modified generalized topological entropy

Koslicki proposed topological entropy [16] of a sequence and defined it as follows:

1 $$ H_{top(\omega)} = \frac{\log_{4}(p_{\omega}(n))}{n}  $$

where the finite DNA sequence has a length of *ω*. Its sub sequence has length of *n*, where 4^*n*^+*n*−1≤|*ω*|≤4^*n*+1^+(*n*+1)−1. And *p*_*ω*_(*n*) is the number of sub sequences of length n within first $\phantom {\dot {i}\!}4^{n_{\omega } }+n_{\omega }-1$ bps of *ω*. In general, topological entropy reflects the complexity and randomness of a sequence. If the sequence has low entropy, it has less randomness. For example, entropy values of exons are supposed to have lower values than that of introns since exons are more conserved and have relatively fixed functions. Moreover, topological entropy is able to compare sequences with different lengths. Generalized topological entropy is a complete form of topological entropy [19] and it is defined as:

2 $$ H_{n_{\omega}}^{k}(\omega) = \frac{1}{k}\sum_{i=n_{\omega}-k+1}^{n_{\omega}} \frac{\log_{4}(p_{\omega}(i))}{i}  $$

where *n*_*ω*_ satisfies $\phantom {\dot {i}\!}4^{n_{\omega }}+n_{\omega }-1 \leq |\omega | \leq 4^{{n_{\omega }}+1}+(n_{\omega }+1)-1$ and *k*≤*n*. *p*_*ω*_(*i*) is the number of different sub sequences within *ω*. Generalized topological entropy includes contributions from all the sub sequences and measures the complexity of DNA sequence more comprehensively. In our method, we modified both topological entropy and generalized topological entropy. Since all sub sequences are counted in entropy calculations, we optimized entropy calculation by filtering out sub sequences which have lower appearance frequencies. The criterion is that if the counting frequency of a sub sequence is smaller than $\phantom {\dot {i}\!}{4^{n_{\omega }}}/{\omega }$, this sub sequence will not be counted in the entropy calculation.

### Genomic signal processing (GSP) and singular value decomposition (SVD)

Genomic signal processing (GSP) based on digital signal processing (DSP) has been used for exon prediction recently. GSP digitalizes DNA sequence and analyze the numerical sequence with different algorithms. We applied GSP method and singular value decomposition (SVD) method to analyze digitalized DNA sequences. In our study, we digitalized sequences by their entropy value and investigated the functional and conserved regions by using SVD method. SVD is a commonly used approach in matrix analysis. Matrix A is decomposed into three matrixes as follows:

3 $$ A_{k \times p} = U_{k \times k} S_{k \times p} V_{p \times p}^{T}  $$

where *U*^*T*^*U*=*I*_*k*×*k*_ and *V*^*T*^*V*=*I*_*p*×*p*_. The columns of *U* are called the left-singular vectors and those of *V* are called the right-singular vectors. *S* is a rectangular diagonal matrix with non-negative real numbers on the diagonal. The diagonal terms *σ*_*i*_ of *S* are the singular values of *S*. And the eigenvalue *λ* of *S* is $\lambda _{i} = \sigma _{i}^{2}$. In some cases, such as when *A* is a sparse matrix, *σ*_*i*_ decrease quickly and *A* can be approximately factorized as:

4 $$ A_{k \times p} \approx U_{k \times r} S_{r \times r} V_{r \times p}^{T}  $$

where *r* is much smaller than *k* and *p*.

## Results

### Modified generalized topological entropy and its application on exploring complexity of exons, introns and promoters

Topological entropy was proposed by Koslicki [16] to solve entropy calculation quest on finite sequences. Generalized topological entropy was proposed by Wang et al. [17] and is a complete form of topological entropy. Both of them can measure complexity of functional elements such as exons and introns in DNA sequence.

In order to highlight the characteristics of repetition sequences, we modified generalized topological entropy and used it to calculate entropy value of exons, introns and promoters on each chromosome in human genome. After we calculated all entropy, mean values of entropy of exons, introns and promoters are listed in Table 1. Meanwhile, the numbers of exons, introns and promoters in each chromosome are also listed in Table 1. We performed a Kruskal-Wallis test to check whether there were significant differences between them (Additional file 1: Table S1).

**Fig. 1:**
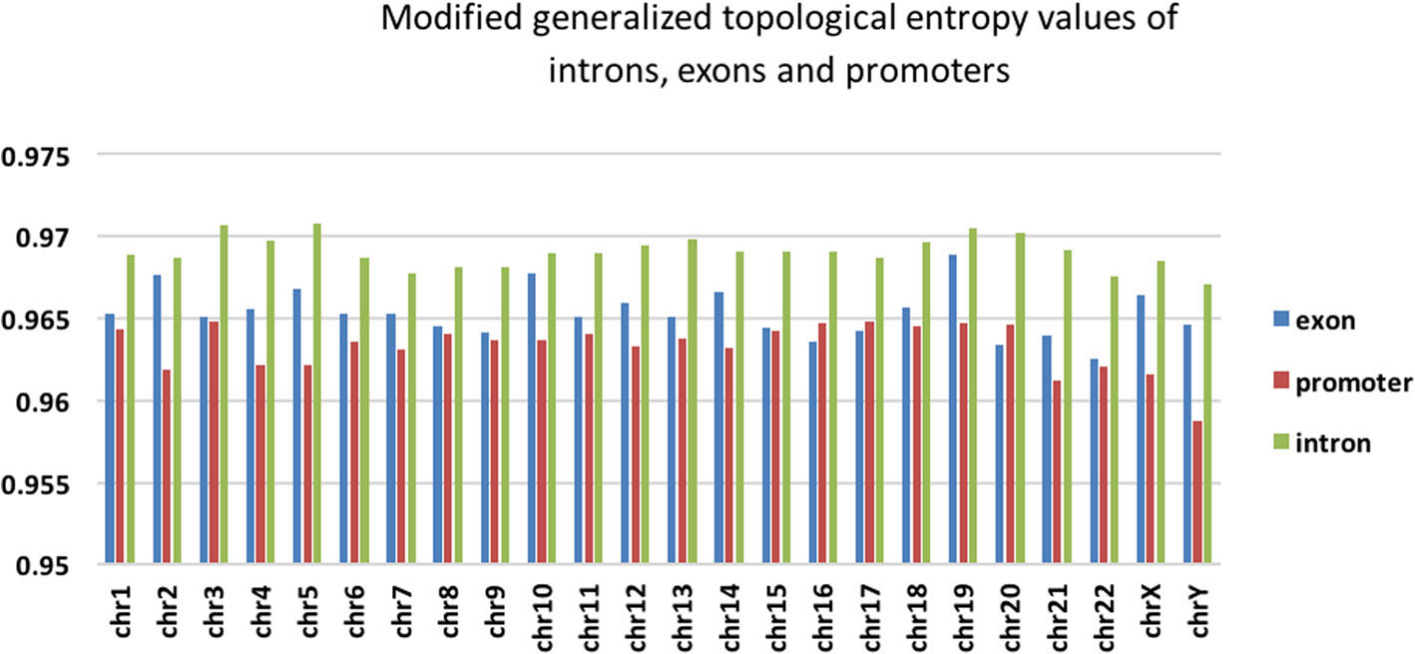
Modified generalized topological entropy values of introns, exons and promoters

We plotted the mean entropy value of exons, introns and promoters on each chromosome in Fig. 1. Figure 1 shows that average modified generalized topological entropy value of exons is lower than that of introns in the same chromosome. The average modified generalized topological entropy value of promoters is lower than that of introns in most chromosomes. Additional file 1: Table S1 shows that differences between exons, introns and promoters are statistically significant.

From the definition of information entropy, a sequence is supposed to have lower entropy value if its sequence is less complex and more conserved. Normally exons are more conserved than introns because they carry more selective pressure in evolution process. Our result shows that entropy value of exon is smaller than that of intron, which is consistent with the theory of evolution. Similarly, promoters are highly conserved elements in DNA sequences [5] and they participate key processed in many living cells, remaining essentially unchanged. Promoter regions often have motifs for binding transcription factors. Our result demonstrates that entropy values of promoters are even smaller than those of exons.

Entropy normally measures the variety of sub-sequences and is not directly related to evolutionary conservation. However, some repetition sequences, which lead to small entropy values, are in conserved regions. For example, some motifs for transcription factors binding in promoters are repetition sequences and they might have regulatory functions. These functions make these regions conserved in evolution.

### Comparison of the generalized topological entropy and modified generalized topological entropy

We calculated the entropy values of exons, introns and promoters in the way of previously reported generalized topological entropy.

Then we tested the differences between exons, introns and promoters in each chromosome. We found that they were significantly different, and the significant level was lower than that calculated by our proposed modified generalized topological entropy (Additional file 1: Table S1). For example, the *p*-value of chromosome 2 is 1.52*e*−14 after we optimized generalized topological entropy calculation, which is less than that from original generalized topological entropy (*p*=7.43*e*−10). It is reasonable since we filtered a small proportion of non-repetitive sequences, highlighted the role of repeated sequences in our modified generalized entropy calculation.

### Entropy-based genomic signal processing analysis with singular value decomposition on a single gene sequence

**Fig. 2:**
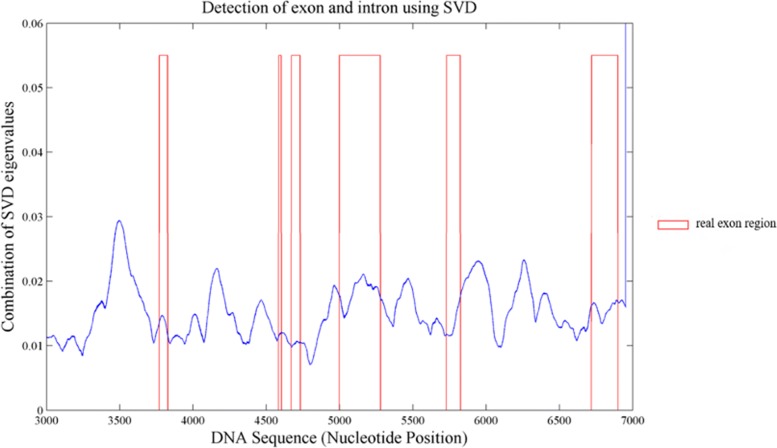
A portion of SVD curve and locations of real exons presented as red box

As mentioned above, topological entropy value can measure the complexity of a sequence region. Therefore, it is used to investigate exon and intron regions on single gene. We applied modified topological entropy calculation to gene AJ229040 with a sliding window size of 100 bps. With *k* value set as 2, 3, 4, 5 and 6, we converted each nucleotide base to a matrix with dimension of 1×5. We then applied SVD approach [33, 34] on the whole numerical gene sequence. A portion of SVD curve along 3000 to 7000bp is shown in Fig. 2 and red boxes indicate real locations of exons.

**Fig. 3:**
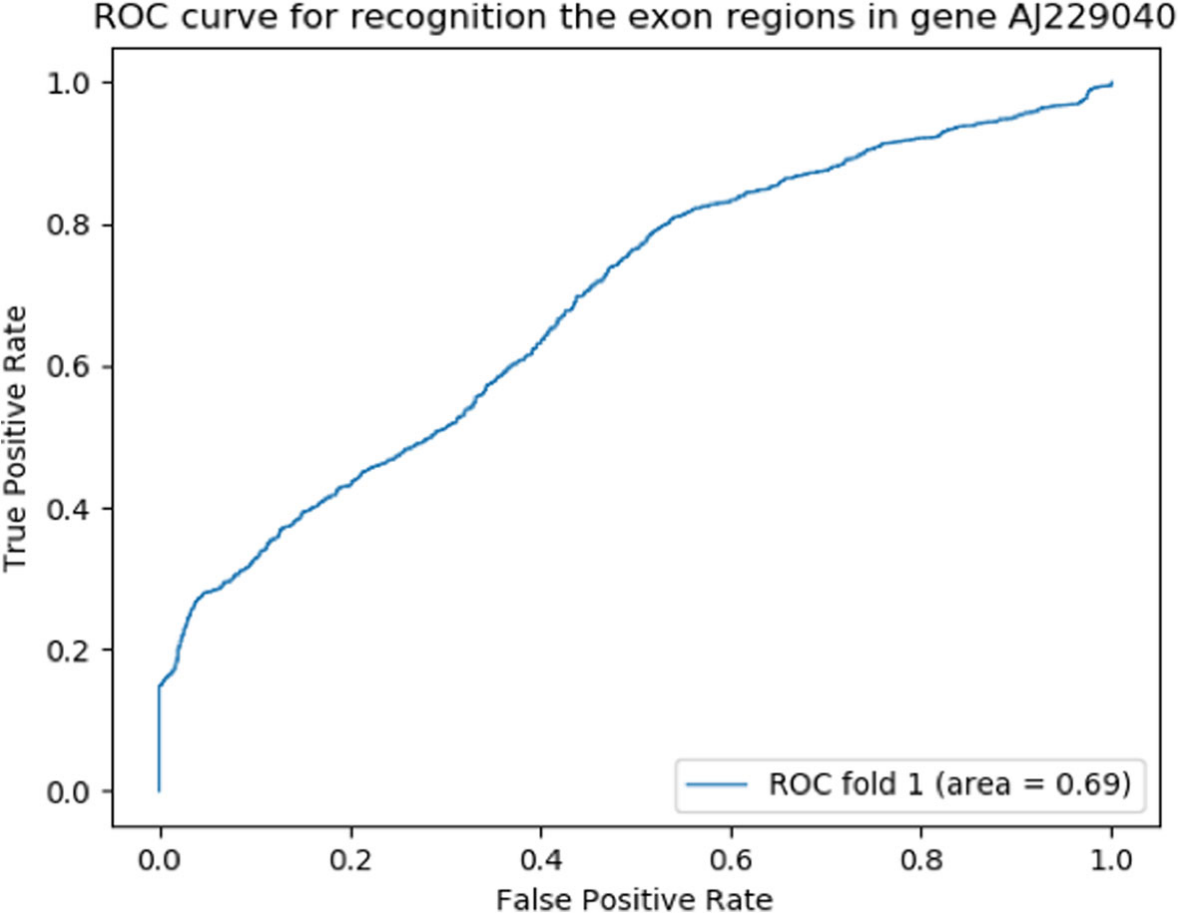
ROC curve for exon and intron prediction in gene AJ229040

We plotted receiver operating characteristic (ROC) curve and choose a cut off value as 0.012 to estimate exon and intron regions. In the region from 3500 bp - 10500 bp, the total length of exons is 688 bps. We correctly predicted 122 exon nucleotides and 4708 introns. The closer the ROC curve is to the upper left corner, the higher the overall accuracy of the prediction. The AUC is a measure of how well a feature can distinguish between exon and intron groups. Without using any prior knowledge, the accuracy of our prediction reaches 0.67 (Additional file 1: Table S2) and the area under the curve (AUC) is 0.69 (Fig. 3).

To assess the feasibility of our approach across different species, we randomly chose some genes from human, dog, zebrafish, C. elegans and fruitfly from Ensembl genome browser [38]. We performed our exon prediction approach (Additional file 1: Table S3) and plotted ROC curves of prediction results for each gene (Additional file 1: Figure S1). It is noted that the AUC value ranges from 0.55 to 0.81. That means our method for exon prediction based on generalized topological entropy is applicable across species.

## Discussion

There exist a large number of exon prediction methods for a single gene or multiple genes [3, 39]. However, most of them highly rely on prior knowledge such as databases of protein-coding genes. These homology-based methods [40, 41] predict genes by comparing sequence with known database sequences. Therefore, it takes much more time to search through the whole database and produce results. These methods [42, 43] have high prediction accuracy in finding homology sequences. However, they have limitations in detecting other functional elements in other 97 – 98% noncoding genomic regions. The rapid development of next-generation sequencing technology leads to big accumulation of omics data. To discover the underlying biological mechanisms from the massive data, homology-based methods are instructive while time consuming. Therefore, more methods such as entropy-based quantitative methods demonstrate their advantages and are applied to analyze various omics data [44–46].

Numerical signal processing approaches are utilized in genomic analysis as genomic signal processing. Generally, information entropy indicates a system status and predicting exon region by just a feature of entropy is a very challenging task. Therefore, we integrated optimized topological entropy and GSP method to calculate complexity of DNA sequences, investigated their application on exon and intron prediction in DNA sequences. We used the same gene which Das and Turkoglu used in their numerical mapping method to predict exons [31]. Their method has higher prediction accuracy than ours since they calculated the entropy based on repetition property of 64 types of codons. Our result is still reasonable since no prior knowledge is used and the prediction only depends on the sequence. That also implies our method to digitalize DNA sequences based on modified generalized entropy is extendable to other element prediction on single sequence or multiple sequences.

To analyze the digitalized DNA sequence, we employed SVD approach in our study. For our case, *U*_*k*×*r*_ in Eq. 4 refers to the highly related entropy calculation modes for different k and $V_{r\times p}^{T}$ represents a series of highly associated nucleotide positions. By using SVD method, correlation information on DNA sequence is investigated and the biological meaning is straightforward. In the future, we will include other methods in this integrated entropy-based GSP approach to improve the result of exon prediction and ROC curve for more species.

## Conclusions

In conclusion, our exon and intron prediction method, which is based on entropy calculation and genomic signal processing, analyzes complexity of exons and introns and is able to distinguish the exon and intron regions across different species. Our research optimizes the existing topological and generalized topological entropy calculation. This integrated approach is extendable to exon and other functional element prediction on the large-scale genome data.

## Additional file

Additional file 1 **Figure S1**. The AUC value of ROC curves from exon prediction results of 15 genes.(a) – (e) The performance obtained by our method for every specie and the value of AUC ranges from 0.55 to 0.81. **Table S1**. Significance level of *p*-value in Kruskal-Wallis Test for generalized topological entropy and modified generalized topological entropy calculation of genetic elements. **Table S2**. Prediction of exon and intron regions on single gene AJ229040. **Table S3**. Prediction of exon and intron regions on genes across five species. (DOCX 17, 765 kb)

## Abbreviations

DNA: Deoxyribonucleic acid GSP: Genomic signal processing
GTE: Generalized topological entropy
MGTE: Modified generalized topological entropy
MTE: Modified topological entropy
SVD: Singular value decomposition
TE: Topological entropy
