# Supplementary material for: Integrated entropy-based approach for analyzing exons and introns in DNA sequences
Source: BMC Bioinformatics. 2019 Jun 10;20(Suppl 8):283. doi: 10.1186/s12859-019-2772-y (PMC6557737; doi:10.1186/s12859-019-2772-y)
Supplement: Supplementary file 1 — Figure S1. The AUC value of ROC curves from exon prediction results of 15 genes.(a) – (e) The performance obtained by our method for every specie and the value of AUC ranges from 0.55 to 0.81. Table S1. Significance level of p-value in Kruskal-Wallis Test for generalized topological entropy and modified generalized topological entropy calculation of genetic elements. Table S2. Prediction of exon and intron regions on single gene AJ229040. Table S3. Prediction of exon and intron regions on genes across five species. (DOCX 17, 765 kb) [file 12859_2019_2772_MOESM1_ESM.docx]

# Integrated entropy-based approach for analyzing exons and introns in DNA sequences Additional File

Junyi Li^1, †, *^, Li Zhang^1, †^, Huinian Li^1^, Yuan Ping^1^, Qingzhe Xu^1^, Rongjie Wang^2^, Renjie Tan^2^, Zhen Wang^3^, Bo Liu^2^ and Yadong Wang^1, 2, *^

^1^School of Computer Science and Technology, Harbin Institute of Technology (Shenzhen), Shenzhen, Guangdong 518055, China

^2^School of Computer Science and Technology, Harbin Institute of Technology, Harbin, Heilongjiang 150001, China

^3^CAS Key Laboratory of Computational Biology, CAS-MPG Partner Institute for Computational Biology, Shanghai Institute of Nutrition and Health, Shanghai Institutes for Biological Sciences, University of Chinese Academy of Sciences, Chinese Academy of Sciences, Shanghai 200031, China

Figure S1. The AUC value of ROC curves from exon prediction results of 15 genes.
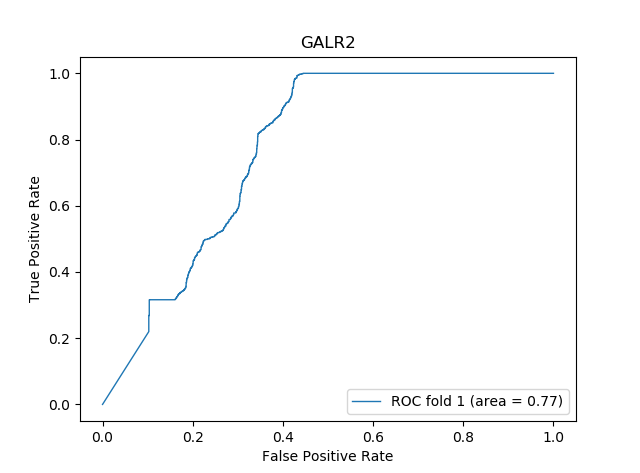

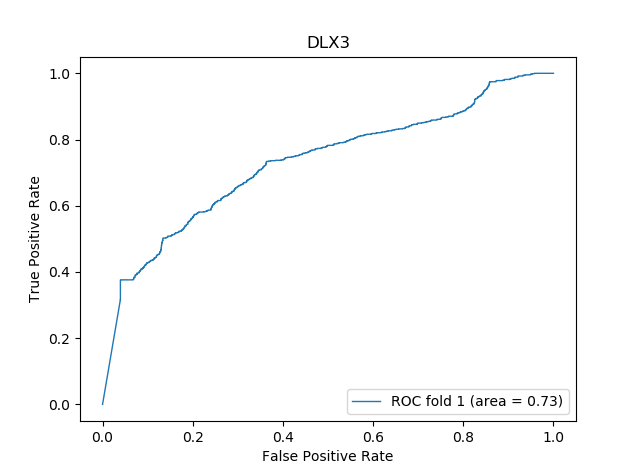
(a) – (e) The performance obtained by our method for every specie and the value of AUC ranges from 0.55 to 0.81.


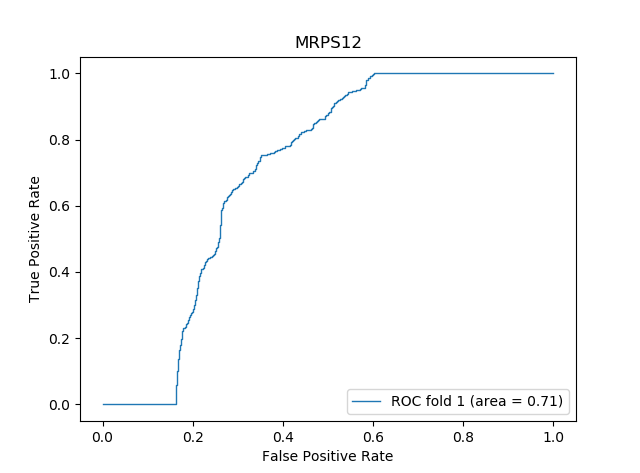


1. ROC curves for gene GALR2, DXL3, MRPS12 in human


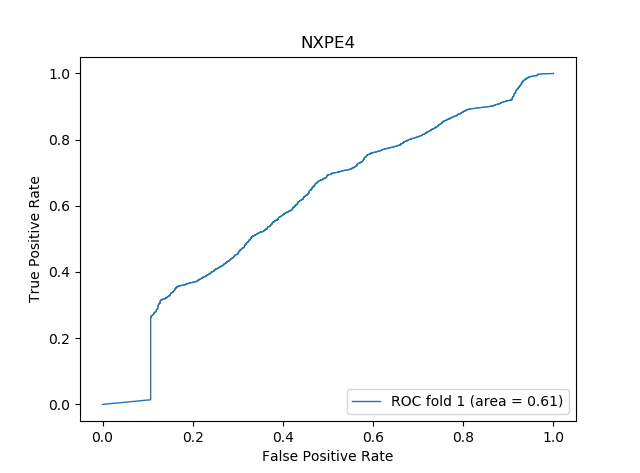

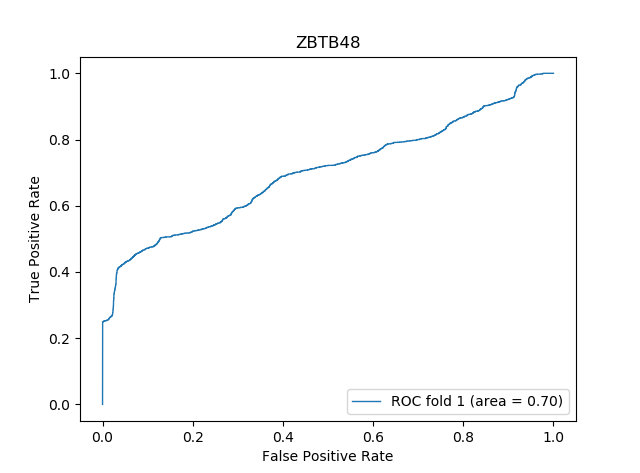


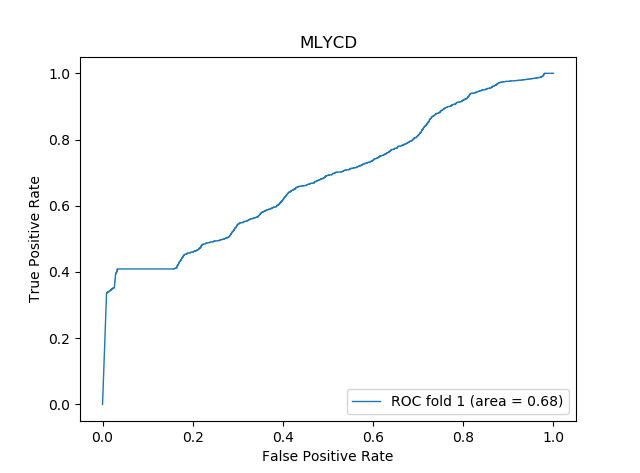


1.
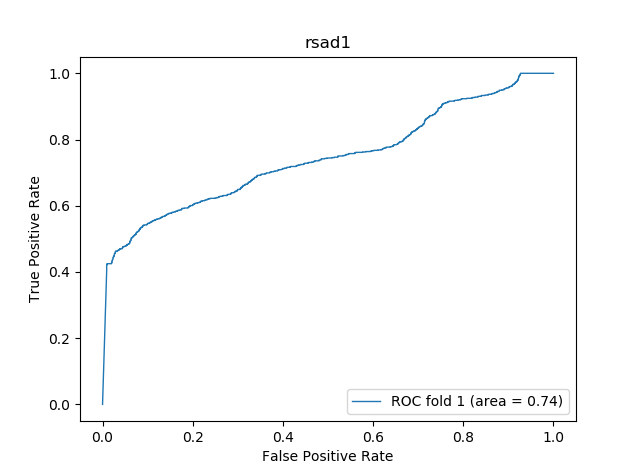
ROC curves for gene ZBTB48, NXPE4, MLYCD in dog


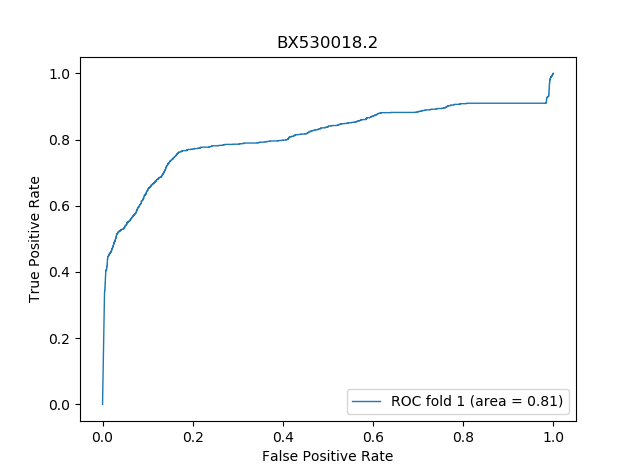


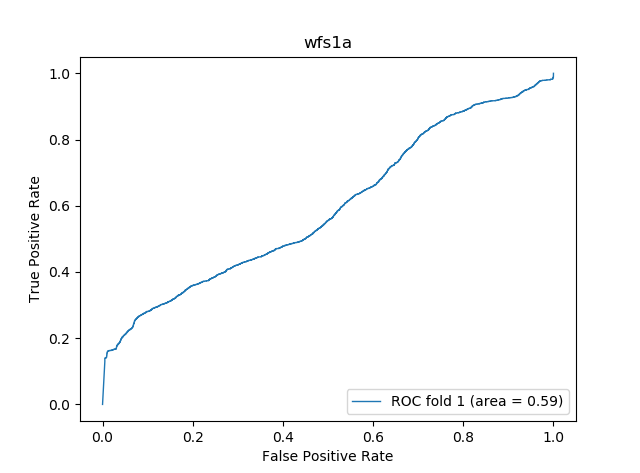


1.
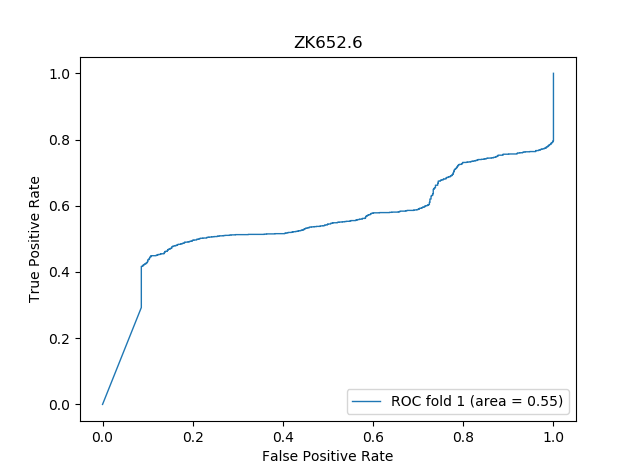

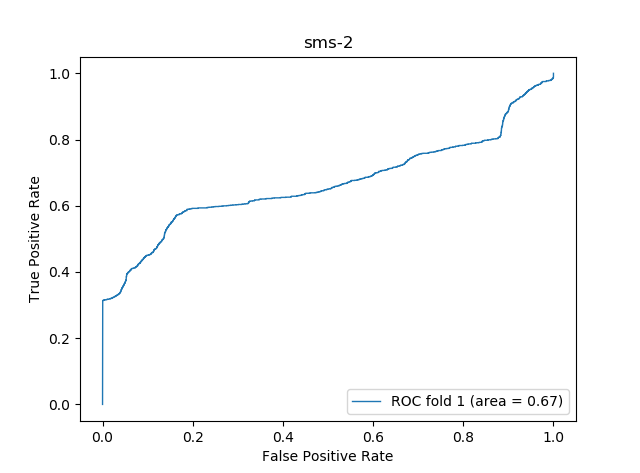
ROC curves for gene rsad1, BX530018.2, wfs1a in zebrafish


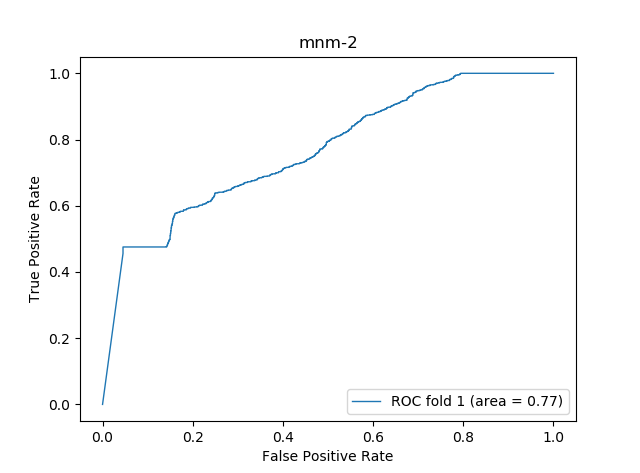


1.
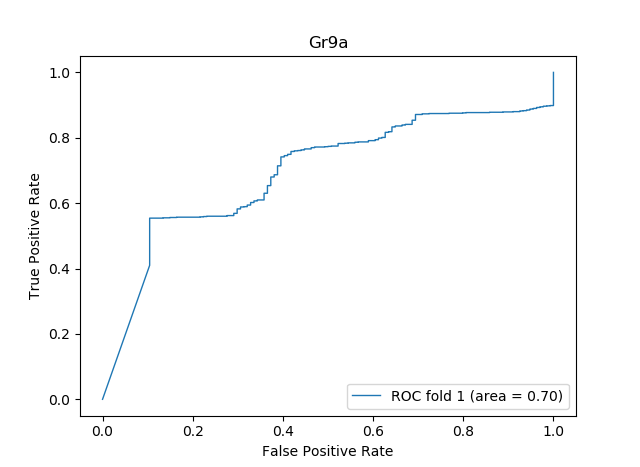
ROC curves for gene sms-2, ZK652.6, mnm-2 in Caenorhabditis elegans


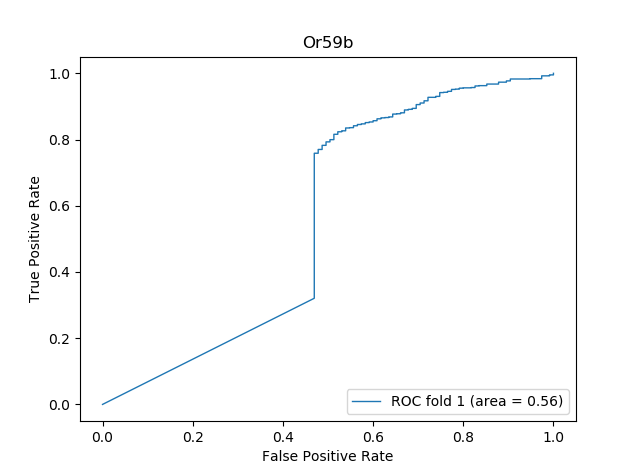


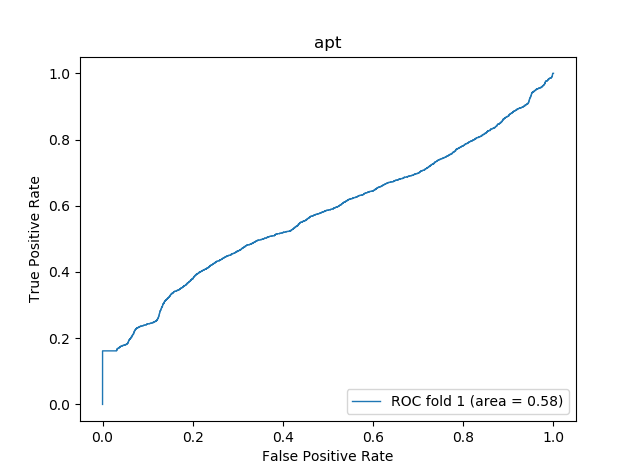


1. ROC curves for gene Gr9a, Or59b, apt in fruitfly

Table S1. Significance level of p-value in Kruskal-Wallis Test for generalized topological entropy and modified generalized topological entropy calculation of genetic elements.

|  | Generalized topological entropy | Modified generalized topological entropy |
| --- | --- | --- |
| chr1 | 1.63E-11 | 1.09E-11 |
| chr2 | 7.43E-10 | 1.52E-14 |
| chr3 | 9.39E-11 | 1.29E-09 |
| chr4 | 9.85E-06 | 1.53E-08 |
| chr5 | 9.14E-06 | 1.85E-13 |
| chr6 | 1.22E-08 | 4.83E-15 |
| chr7 | 1.58E-09 | 2.20E-11 |
| chr8 | 8.40E-07 | 9.02E-15 |
| chr9 | 1.33E-09 | 2.27E-13 |
| chr10 | 2.41E-06 | 2.76E-14 |
| chr11 | 2.05E-08 | 2.21E-15 |
| chr12 | 3.36E-06 | 1.67E-10 |
| chr13 | 5.86E-08 | 6.23E-12 |
| chr14 | 2.23E-09 | 1.27E-09 |
| chr15 | 4.95E-08 | 2.65E-11 |
| chr16 | 3.14E-11 | 1.19E-13 |
| chr17 | 3.83E-08 | 9.24E-13 |
| chr18 | 4.17E-07 | 1.15E-08 |
| chr19 | 1.42E-09 | 8.30E-14 |
| chr20 | 1.13E-09 | 1.97E-12 |
| chr21 | 7.86E-06 | 6.06E-16 |
| chr22 | 3.55E-10 | 2.90E-10 |
| chrX | 5.16E-08 | 1.07E-10 |
| chrY | 3.38E-08 | 1.88E-14 |

Table S2. Prediction of exon and intron regions on single gene AJ229040.

|  | Value |  |
| --- | --- | --- |
| TP | 122 |  |
| TN | 4708 |  |
| FP | 1604 |  |
| FN | 566 |  |
| Acc | 0.69 |  |
| Sn | 0.177326 |  |
| Sp | 0.070684 |  |
|  |  |  |
| TP: True exon region | | |
| FP: False exon region | | |
| TN: True non-exon region | | |
| FN: False non-exon region | | |
| Acc: Accuracy | | |
| Sn: Sensitivity | | |
| Sp: Specificity | | |

Table S3. Prediction of exon and intron regions on genes across five species.

Human：

| Gene | GALR2 | DLX3 | MRPS12 |
| --- | --- | --- | --- |
| cutoff | 0.015 | 0.013 | 0.013 |
| TP | 796 | 91 | 358 |
| FP | 1625 | 705 | 2651 |
| TN | 247 | 3006 | 539 |
| FN | 368 | 773 | 59 |
| Acc | 0.34 | 0.68 | 0.25 |
| Precision | 0.33 | 0.11 | 0.12 |
| Sn | 0.68 | 0.11 | 0.86 |
| Sp | 0.13 | 0.81 | 0.17 |
| AUC | 0.77 | 0.73 | 0.71 |
| exon number | 2 | 3 | 2 |
| exon length | 3036 | 4575 | 3607 |

Dog：

| Gene | ZBTB48 | NXPE4 | MLYCD |
| --- | --- | --- | --- |
| cutoff | 0.016 | 0.012 | 0.013 |
| TP | 48 | 103 | 17 |
| FP | 293 | 363 | 362 |
| TN | 4329 | 19107 | 12490 |
| FN | 2027 | 1528 | 1479 |
| Acc | 0.65 | 0.91 | 0.87 |
| Precision | 0.14 | 0.22 | 0.04 |
| Sn | 0.02 | 0.06 | 0.01 |
| Sp | 0.94 | 0.98 | 0.97 |
| AUC | 0.70 | 0.61 | 0.68 |
| exon number | 11 | 5 | 6 |
| exon length | 6697 | 21101 | 14348 |

Zebrafish:

| Gene | rsad1 | BX530018.2 | wfs1a |
| --- | --- | --- | --- |
| cutoff | 0.013 | 0.012 | 0.012 |
| TP | 32 | 130 | 53 |
| FP | 976 | 142 | 184 |
| TN | 9498 | 7439 | 15559 |
| FN | 789 | 1309 | 2638 |
| Acc | 0.84 | 0.84 | 0.85 |
| Precision | 0.03 | 0.48 | 0.22 |
| Sn | 0.04 | 0.09 | 0.02 |
| Sp | 0.91 | 0.98 | 0.99 |
| AUC | 0.74 | 0.81 | 0.59 |
| exon number | 6 | 8 | 7 |
| exon length | 11295 | 9020 | 18434 |

Caenorhabditis elegans:

| Gene | F53H8.4 | ZK652.6 | C10A4.8 |
| --- | --- | --- | --- |
| cutoff | 0.013 | 0.015 | 0.013 |
| TP | 283 | 323 | 77 |
| FP | 322 | 37 | 441 |
| TN | 2287 | 777 | 881 |
| FN | 1166 | 1044 | 842 |
| Acc | 0.63 | 0.50 | 0.43 |
| Precision | 0.47 | 0.90 | 0.15 |
| Sn | 0.20 | 0.24 | 0.08 |
| Sp | 0.88 | 0.95 | 0.67 |
| AUC | 0.67 | 0.55 | 0.77 |
| exon number | 6 | 6 | 6 |
| exon length | 4058 | 2181 | 2241 |

Fruitfly:

| Gene | Gr9a | Or59b | apt |
| --- | --- | --- | --- |
| cutoff | 0.014 | 0.014 | 0.012 |
| TP | 4 | 96 | 297 |
| FP | 117 | 34 | 1158 |
| TN | 4505 | 81 | 14453 |
| FN | 2071 | 952 | 2529 |
| Acc | 0.67 | 0.15 | 0.80 |
| Precision | 0.03 | 0.74 | 0.20 |
| Sn | 0.00 | 0.09 | 0.11 |
| Sp | 0.97 | 0.70 | 0.93 |
| AUC | 0.70 | 0.56 | 0.58 |
| exon number | 3 | 3 | 5 |
| exon length | 6697 | 1163 | 18437 |
